# Awareness and attitudes towards dental implants among dental patients in India: A questionnaire-based survey

**DOI:** 10.6026/973206300211351

**Published:** 2025-06-30

**Authors:** Monal Karkar, Rik Chatterjee, Shashidevi Haranal, Jasleen Kaur, Nitesh Naresh, Nimish Nimeya, Ritik Kashwani

**Affiliations:** 1 Department of Oral and Maxillofacial Surgery, Rural Dental College, Pravara Institute of Medical Sciences, Loni, Maharashtra, India; 2 Department of Prosthodontics and Crown & Bridge, Sri Aurobindo College of Dentistry, Indore, Madhya Pradesh, India; 3 Department of Oral and Maxillofacial Surgery, ITS Dental College, Hospital and Research Centre, Greater Noida, Uttar Pradesh, India; 4 Department of Dentistry, Pt. Bhagwat Dayal Sharma, Institute of Dental Sciences, Rohtak, Haryana, India; 5 Department of Dentistry, Army College of Medical Sciences, Delhi Cantt, New Delhi, India; 6 Department of Periodontology, PDM University, Bahadurgarh, Haryana, India; 7 Department of Oral and Maxillofacial Radiology, School of Dental Sciences, Sharda University, Greater Noida, Uttar Pradesh, India

**Keywords:** Awareness, attitudes, dental implants, patient education, questionnaire

## Abstract

Dental patients' awareness and impressions on dental implants is of interest using a standard questionnaire. Out of the three hundred individuals, 48% correctly identified dental implants as a fixed prosthesis replacing a tooth root and 72% had heard of them. Despite obstacles, including significant cost, anxiety and ignorance, still exist, most respondents had positive impressions; 66% of them highly rated dental implants. Strongly correlated were higher degrees of education and growing acceptance of dental implants, as well as awareness. These results highlight the need for improved patient education and focused communication techniques to raise acceptability and informed decision-making about dental implant treatments.

## Background:

Considered a significant advancement in the field of dentistry, dental implants offer a practical and long-lasting substitute for missing teeth [1]. The rising interest in dental implants worldwide can be explained in part by their natural appearance, longevity and the improved quality of life they provide for patients. By replacing more conventional techniques, such as bridges or dentures, dental implants enable patients to restore the functional and cosmetic aspects of their smile, thereby transforming oral rehabilitation [2]. Although dental implants have numerous advantages and efficacy, knowledge of them among patients still generates concerns in various fields. It is essential to understand how dental patients perceive dental implants and what factors influence their acceptance decision [3]. In a basic sense, information about dental implants significantly influences patient decisions. Ignorant of the advantages of modern technology connected with dental implants, patients may be reluctant to give them any thought. Furthermore, ignorance of the operations needed, the expenses and the long-term effects of implants could result in false knowledge and misinterpretation. These difficulties could force patients to choose less ideal options, such as bridges or dentures, therefore influencing their general degree of satisfaction and quality of life [4].

Conversely, understanding of dental implants is linked with increased acceptability, especially when patients are aware of the expected benefits, including improved function, comfort, aesthetics and speech [5]. Apart from awareness, the attitude of dental patients significantly influences their readiness for dental implant treatment. Views on pain, faith dental practitioners, concerns about the expense and general judgments on current medical achievements varies significantly [6, 7]. Furthermore, characterizing these attitudes in part involves psychological, cultural, financial and pragmatic aspects. Higher socioeconomic level patients, for example, might see the costs and benefits of dental implants more favourably than those from lower income levels would find to be financially unacceptable. Treatment decisions also rely on cultural perspective; some people could support traditional methods either out of ignorance or cultural beliefs about dental procedures [8, 9]. This study is to assess, among dental patients, their awareness and attitudes about dental implants utilizing a questionnaire-based survey. By evaluating the degree of awareness, the variables affecting patient attitudes and their whole impressions of dental implants, this study aims to identify possible areas for advancement in patient education and communication tactics. It will also help to clarify the challenges patients have choosing dental implants, thereby allowing dental practitioners to change their approach to treatment and counselling. Encouragement of the general acceptance of dental implants depends on an understanding of these aspects, which also guarantees that patients choose wisely, depending on reliable knowledge [10]. Therefore, it is of interest to report the awareness and attitudes towards dental implants among dental patients in India using a questionnaire-based survey.

## Methodology:

This study aimed to evaluate dental patients' awareness and opinions of dental implants through a questionnaire-based survey. The study design, sample selection, data collection technique and statistical analysis applied to investigate the responses are discussed, along with the methodology employed.

## Study design:

This was a cross-sectional descriptive study designed to gather data from dental patients visiting a dental clinic for either general or specialty treatment. The survey inquired about their impressions, knowledge and opinions on dental implants. The research method provided a snapshot of public ideas and expertise at a given moment, thereby revealing current trends.

## Population and sample selection:

The target population for this study consisted of dental patients who visited dental clinics in a specific geographical area, primarily urban and suburban settings. Participants were selected based on the following inclusion and exclusion criteria:

## Inclusion criteria:

[1] Patients aged 18 and above.

[2] Both genders.

[3] Patients who received routine dental care or consultations, regardless of their specific reason for visit.

[4] Patients who could provide informed consent to participate in the survey.

## Exclusion criteria:

[1] Patients who were not capable of understanding or responding to the survey (*e.g.*, due to language barriers, cognitive impairments, or illiteracy).

[2] Patients under 18 years of age.

[3] Individuals who had already received dental implants (as they may have had different perspectives compared to those who were considering implants).

A simple random sampling technique was used to select participants from a list of patients who met the inclusion criteria. The study aimed for a sample size of 300 participants, based on standard recommendations for survey-based research, which ensured sufficient statistical power to detect meaningful patterns and relationships in the data.

## Data collection method:

The data were collected using a structured questionnaire designed to measure awareness and attitudes towards dental implants. The questionnaire consisted of two main sections:

## Section 1:

## Demographic information:

This section collected basic demographic data, including age, gender, education level, socioeconomic status and dental history.

## Section 2:

## Awareness and attitudes towards dental implants:

This section assessed the participants':

## Awareness:

Their knowledge of dental implants, include understanding of the procedure, benefits, risks and costs. It included questions such as "Had you heard of dental implants?" and "What did you know about the benefits of dental implants?"

## Attitudes:

Their perceptions of dental implants include their perceived advantages, concerns (*e.g.*, cost, pain, or aesthetic outcomes) and willingness to consider implants as a treatment option. For instance, "Would you have considered dental implants if you lost a tooth?" and "What concerns did you have about getting a dental implant?" A small sample of twenty people pre-tested the questionnaire to ensure its dependability and clarity. Depending on patient preferences, the questionnaire was then sent to the chosen sample of participants either personally or via an online survey platform, once it had been improved based on the comments.

## Data analysis:

The collected data were analyzed using quantitative methods. The statistical software SPSS (Statistical Package for the Social Sciences) was used to conduct the analysis. The following steps were taken for data analysis:

## Descriptive statistics:

The demographic characteristics of the participants were summarized using frequencies, percentages and means.

## Awareness analysis:

The participants' awareness of dental implants was assessed by the number of correct responses to knowledge-based questions, resulting in a composite awareness score.

## Attitude analysis:

Participants' attitudes were evaluated using a Likert-scale response format, with options including "Strongly Agree," "Agree," "Neutral," "Disagree," and "Strongly Disagree." The responses were analyzed to identify common themes in patient attitudes.

## Cross-tabulation:

Cross-tabulation analysis was performed to identify relationships between demographic variables (age, gender, education level, *etc.*) and awareness or attitudes towards dental implants.

## Chi-square test:

Chi-square tests were used to determine if there were significant differences in awareness and attitudes across different demographic groups (*e.g.*, age, gender and education).

## Reliability analysis:

Cronbach's Alpha was used to test the internal consistency of the survey items related to attitudes and awareness.

## Ethical considerations:

The study adhered to ethical guidelines to ensure the protection of participants' rights and privacy. The following steps were implemented:

## Informed consent:

All participants were informed about the purpose of the study, their voluntary participation and the confidentiality of their responses. Written informed consent was obtained from each participant before their involvement.

## Confidentiality:

Personal information was kept confidential and stored securely. No identifying information was included in the study results.

## Ethical approval:

Ethical approval was sought from the institutional review board (IRB) or ethics committee of the dental institution conducting the study.

## Results:

Table 1 (see PDF) presents the results of Knowledge, Attitudes and Practices (KAP) questionnaire on dental implants, providing a comprehensive overview of respondents' demographics, awareness and practices related to dental implants. The majority of respondents (48%) were in the age group of 25-34 years, with significant portions also in the 45-54 and 35-44 age ranges. Males made up 52% of the respondents and 44% were female. In terms of education, 40% of respondents had completed college or university, while 28% had only completed primary or secondary school, indicating a reasonably educated sample. When asked about dental implants, 72% of respondents had heard of them, with 48% correctly identifying them as a fixed prosthesis to replace a missing tooth root. Additionally, 40% knew that titanium is a commonly used material for dental implants.

Most respondents (40%) believed that dental implants last between 10 and 15 years and the attitudes towards dental implants were generally positive, with 66% rating their opinion as upbeat or very optimistic. However, despite this favorable view, 32% of respondents preferred alternatives such as dentures or bridges, with cost, fear of the procedure and lack of knowledge also being significant barriers. When it comes to recommending dental implants, 60% would endorse them, while 20% would not, showing a relatively high level of confidence in dental implants as a treatment option.

## Discussion:

The analysis of the KAP questionnaire data regarding dental implants aligns with findings from several previous studies that have examined similar trends in various global contexts. Most respondents in the current study were aware of dental implants, with 72% reporting prior knowledge, which is consistent with a study conducted by Ali *et al.* (2023) in Riyadh, Saudi Arabia [10]. Their research found that awareness and knowledge of dental implants were positively correlated with education and socio-economic status, which mirrors the demographic profile observed in the current data, where a significant proportion of respondents had a college or university education. When it comes to understanding what a dental implant is, the current study revealed that 48% correctly identified it as a fixed prosthesis to replace a missing tooth root. This finding aligns with those from a similar study by Mishra *et al.* (2015) [11], which reported a relatively high level of understanding of dental implants among patients. The materials commonly used for dental implants, particularly titanium, were identified correctly by 48% of the respondents, reflecting knowledge congruent with findings by Kohli *et al.* (2015) [12], where titanium was also the most commonly recognized material. However, misconceptions about gold and ceramic materials were prevalent, which aligns with gaps in knowledge observed in other global studies.

The longevity of dental implants, with 40% of respondents believing they last 10-15 years, is consistent with findings from Chihnitha *et al.* (2024) [13], where a significant portion of participants also indicated a similar lifespan. In terms of attitudes, 66% of respondents in the current study had an optimistic or very optimistic view of dental implants, which is consistent with several studies that found favorable attitudes toward dental implants. However, a survey by Chaudhary *et al.* (2015) [14] in India found that while awareness was high, financial constraints and fear of the procedure were significant deterrents. This finding is corroborated in the current study, where 32% of respondents expressed a preference for alternative solutions, such as dentures or bridges and 24% cited the high cost of implants as a primary reason for not choosing them. Finally, the recommendation rate of 60% aligns with findings from Deeb *et al.* (2017) [15] and Hosadurga *et al.* (2007) [16], where a majority of patients and professionals believed in recommending implants, albeit with caution depending on individual circumstances.

## Conclusion:

We show that the knowledge, attitudes and practices surrounding dental implants are consistent with previous research, highlighting both positive perceptions and significant barriers related to cost, fear and medical considerations. These findings suggest the need for continued public education to address misconceptions and barriers and for dental professionals to emphasize the long-term benefits of dental implants while providing clearer information on their cost and safety.
